# Connect-ROD – development and qualitative evaluation of a community-based group intervention to support well-being in patients with a rare or orphan disease

**DOI:** 10.1186/s13023-024-03252-4

**Published:** 2024-07-05

**Authors:** Cécile Bardon, Marie-Éve Rioux, Mélina Rivard, Floryana-Maria Viquez Porras, Julie Zaky

**Affiliations:** Psychology Department, Université du Québec À Montréal (UQAM), 100 Sherbrooke Ouest, Montréal, Québec H2X 3P2 Canada

**Keywords:** Rare disease, Orphan disease, Psychological intervention, Group intervention, Program development, Program evaluation, Acceptance and commitment therapy

## Abstract

**Background:**

Patients living with various rare or orphan diseases (ROD) experience common psychosocial difficulties. Those need emerge from a combination of factors, such as the large variety of patients and the rarity of resources, as well as concentrated efforts on physical health needs that yielded increases in life expectancy and quality in patients. A gap is therefore rising in the consideration of psychosocial needs of patients, such as coping with the impacts of physical limitations, reducing social isolation and distress. To contribute to address this gap, we developed, pilot-tested and evaluated the acceptability, feasibility, implementation, and short-term effects of Connect-ROD, an online group intervention to support adult patients with a ROD (AP-ROD), which aims to improve coping mechanisms, reinforce sense of control, and support personal goals of AP-ROD.

A qualitative study comprising of in-depth pretests, post-test interviews and standardized questionnaires, was conducted with 14 participants in two consecutive intervention groups.

**Results:**

The Connect-ROD intervention is strongly anchored in acceptance and commitment therapy as well as community psychology approaches. A pilot test allowed us to improve on the initial structure and to produce a manualized 10-week program delivered online, made up of 2-h sessions comprising formal activities, exchanges and homework. The evaluation showed satisfactory acceptability and accessibility, compliant delivery by facilitators, and promising short-term effects on personal objectives, sense of control, coping mechanisms, symptom management, acceptance of the emotions associated with the disease, distress, self-efficacy, social support and connectedness. The program did not show short-term effects on overall quality of life.

**Conclusion:**

It is recommended that Connect-ROD is evaluated on a larger scale. It seems promising to support various AP-ROD who live with the complex psychosocial consequences of their disease.

## Background

Rare diseases are defined by their low prevalence, affecting less than 1 in 2000 people (there are between 7000 and 8000 identified rare diseases) and orphan diseases do not have clear effective or promising treatments [1].Despite major differences in symptoms, limitations, treatments and medical needs, patients with rare or orphan diseases (ROD) experience many of the same realities including limited access to services and psychological difficulties rarely acknowledged in services [2, 3]. ROD also share a critical lack of information and adapted resources regarding etiology, diagnosis and treatment for patients, families and health professionals. Consequently, patient populations with ROD have common psychosocial needs and deal with complex health care trajectories. They also contend with common limitations and disabilities, as well as common health problems, such as degenerative and critical health processes, all of which could be considered independently from specific diseases [2, 3]. The quality of life of patients with ROD is lower than for the general population in three key areas: physical limitations (movements, pain, sleep problems, attention or concentration problems that affect daily life), [3–9], social isolation and stigmatization [3, 7, 9–13], and emotional distress (anger, depression, anxiety, hopelessness, loss, lack of control, abandonment) [3, 5, 8, 14–17].

Few studies have addressed the psychosocial interventions needed to support patient populations with ROD, even if the need to develop and evaluate such support and studies have been repeatedly highlighted [18]. With the aim of contributing to fill this gap, this article details how we developed an online group intervention to support adult patients living with a ROD (AP-ROD), and pilot-tested and evaluated its acceptability, feasibility, implementation, and short-term effects. The intervention focuses on helping AP-ROD to improve coping mechanisms, reinforce their sense of control and support their personal goals.

### Evidence for combined programs to support well-being in AP-ROD

In a recent critical literature review, Bardon, Guillemette [18] found that programs combining multiple approaches, such as cognitive behavioural therapy (CBT), acceptance and commitment therapy (ACT), psychoeducation and physical activation, seem promising for AP-ROD. All these approaches seem to increase adaptation, social functioning, and quality of life (QOL), while reducing distress and symptoms through the understanding and treatment of current problems. They also emphasized connecting context with cognitions and emotions, as well as working on motivation, social support, and cognitive restructuring of intrusive or damaging thoughts. Programs based on a CBT approach seem to help reduce distress symptoms and cognitive dysfunctions, improve pain management, and increase well-being [19–21]. Interventions based on ACT seem to help improve QOL and self-management [22]. Both physical activity and psychoeducation programs seem to help reduce distress in AP-ROD [23, 24]. In addition, programs comprising of multiple approaches have a positive though small effect on patients’ distress and QOL. ACT approaches seem particularly well suited to help patients learn to live with the emotional impact of unchangeable medical conditions, especially when paired with complementary interventions.

The mode of program delivery for these structured clinical interventions have been evaluated in individual and group settings. Bardon, Guillemette [18] indicate that group delivery and individual intervention can achieve similar results with AP-ROD. Groups can be community or web-based and, therefore, can be organized outside of hospital settings and in the community [25].

Current studies have not reached a consensus on the difference of face-to-face versus online delivery of psychosocial interventions for patients dealing with chronic, severe, rare, or orphan diseases. Although some research seems to indicate that programs carried out in person can be marginally more effective than online [26–28], taking part in a program over the internet may present some benefits for patients. For example, Web-based programs may be particularly relevant to patients who live in remote areas or who are limited in their movements [18].

Since communication technologies are rapidly evolving, along with patients’ familiarity with their use, it will be relevant to draw from the most recent and upcoming experiences in this field.

### A new group intervention for adults patients living with a rare or orphan disease (Connect-ROD)

When it comes to medical and psychological support and care, AP-ROD face numerous obstacles as well as a generalized lack of resources. Patients experience social isolation and often have difficulty finding a support community because of the rarity of their condition [3, 10, 11]. Group therapy may meet several patients’ needs, such as reducing social isolation and allowing patients to share their experience, while also improving access to adequate psychosocial support and care [18].

**Fig. 1:**
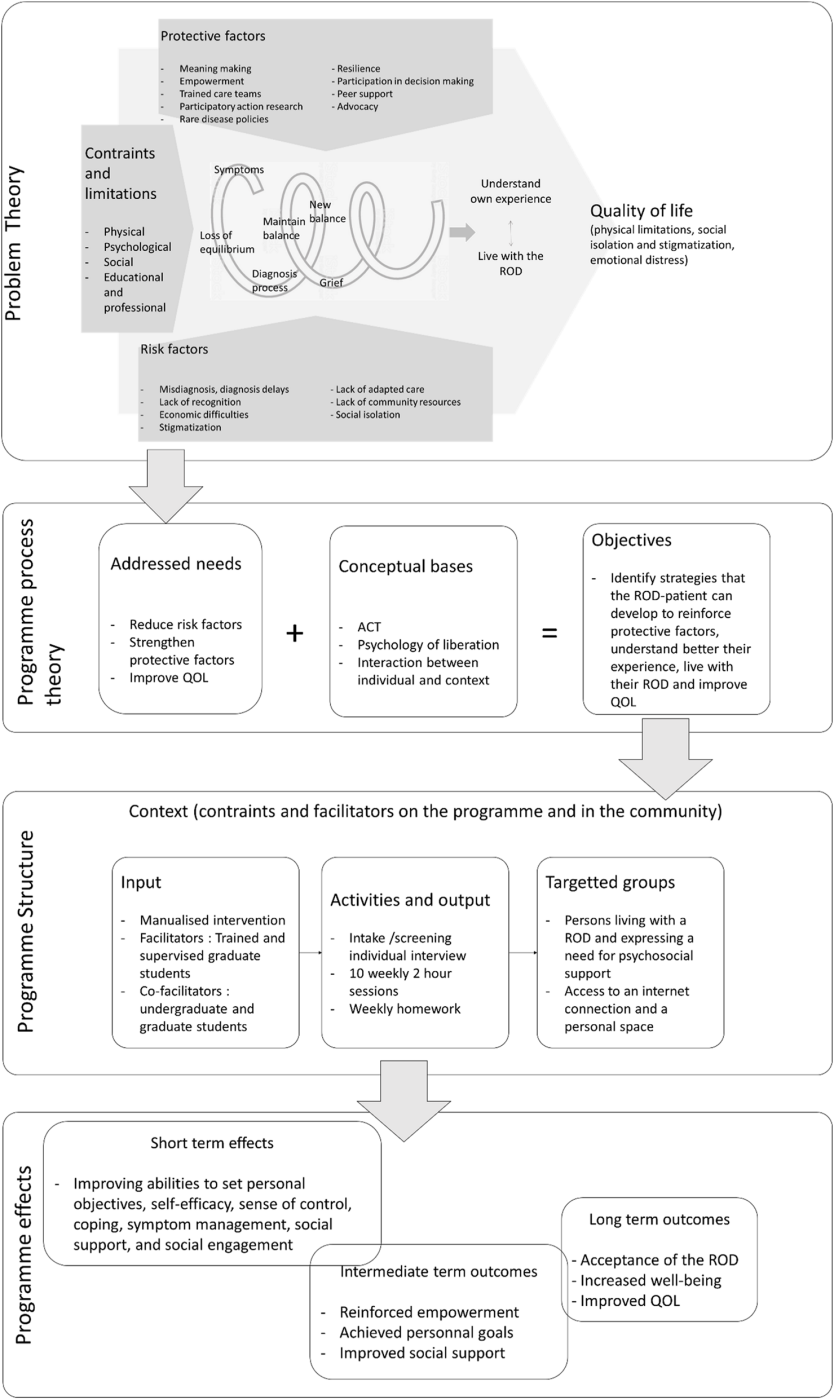
Logic conceptual and operational model for Connect-ROD

In this context, we conducted two preliminary phases: (1) an extensive evaluation of the psychosocial needs of AP- ROD [29]; and (2) an in-depth analysis of current best practices for group interventions, as to identify evidence-based and promising components of group programs for AP-ROD [18]. The current phase developed a group intervention for AP-ROD (Connect-ROD). The theoretical logic model of Connect-ROD is based on programme theory driven evaluation as described by Chen [30] and illustrated in Fig. 1. The program was pilot tested in 2020 (Group 1, *N* = 4 patients) and evaluated with Groups 2 (*N* = 6) and 3 (*N* = 8, 2021, Table 1). It was developed from our preliminary phases (needs analysis and scoping review) and by a team of clinicians specializing in ACT and in community psychology (coauthors FV, MR).

**Table 1:** Participation process and measurement times (G2, G3)

	timeframe	Inception interview for Connect ROD (week -2)	Recruitment for the evaluation (weeks -2 and -1)	Pretest (interview + questionnaire, week -1)	Connect-ROD (Weeks 1 -10)	Attrition in Connect ROD program	Post-test (interview, weeks 11–12)	Follow-up (questionnaire, weeks 14–16)
G1	Fall 2020				4	1	4	
G2	Winter 2021	6	6	6	6	0	6	6
G3	Spring 2021	10	8	8	10	3	8	6
Total		16	14	14	20	4	18	12

Table 1 describes the participation process for the three groups of persons who received the programme during the study. It includes participation in the programme (*N* = 20) and in the evaluation study (*N* = 18). Since the four participants in group 1 were interviewed at post test, they are included in the table. *N* = 14 participants from groups 2 and 3 participated in the pretest, posttest and follow-up leg of the project. Attrition is also indicated for both programme (*N* = 4) and evaluation (*N* = 2)

As described in Fig. 1, Connect-ROD aims to support AP-ROD through group processes, to identify strategies to improve their QOL and to reduce the psychosocial impacts of the disease. More specifically, it aims to improve coping mechanisms, reinforce a sense of control and empowerment, and support the definition and achievement of personal goals.

The program is grounded in three complementary theoretical approaches that were chosen because they showed promising results in group settings with patients dealing with chronic, severe, rare or orphan diseases [18], specifically:

- Acceptance and commitment therapy (ACT) has shown promising results in improving the well-being and individual empowerment of ROD-patients [18]. This third wave cognitive behavioural therapy is sensitive to the context and functions of psychological processes, and emphasizes contextual and experiential change strategies. It aims to develop large, flexible, effective mechanisms to cope with complex issues, within a pragmatic intervention [31]. This approach can be particularly relevant with patients experiencing a complex, multidetermined situation they can barely change, such as ROD-patients.
- Community psychology [32] is a perspective in psychology that aims to understand individuals’ well-being within an ecological framework, considering power iniquities (i.e. between a diseased person and the health system), social inequalities in health (i.e. understand the impact of both poverty and disease on the person), marginalisation (i.e. understand the impact of discrimination on stress) or intersectionality (i.e. take into account the exponential combined impact of disability, gender and race). A community psychology approach of group intervention for AP-ROD allows us to consider patients in their social, cultural and medical contexts, to work with them and their surroundings on their individual and collective empowerment, and to support their autonomy.
- Psychology of liberation [33] can be approached via a community psychology perspective. It takes its roots in Latin-American resistance and social sciences. It is a process of transforming the conditions of inequality and oppression and the institutions and practices that produce them. It is both collective and individual. It aims to support empowerment, the development of new forms of social identities, political conscientization, activism and social change [34]. In the context of the Connect-ROD program, empowering marginalized patients to understand the social institutions and processes that contribute to their stress is crucial. Supporting patients in the development of individual and collective change is also important. This approach is particularly relevant with ROD patients who are often activists in their field [35].

The program is facilitated by a trained professional in ACT (psychologist, social worker, psychoeducator) supported by a co-facilitator (peer-support ROD patient or trainee). It is composed of 10 weekly two-hour sessions that combine elements of psychoeducation, individual as well as group work based on narrated metaphors, images or videos, homework, meditation, a meeting with a health professional working in specialized services for AP-ROD to discuss specific needs for psychosocial support, and the production of a message for future groups of participants. All 10 sessions are structured the same way, with an introduction to the session’s theme and key concepts, a brief overview of theories associated with the theme (i.e. cognitive defusion), a mindfulness exercise to ground participants in the moment and the session, a series of two to three group activities based on manualised ACT programs, designed to practice the learned concepts (i.e. reading and discussing the metaphor of the white page to apply cognitive defusion) or on psychology of liberation (i.e. recognising contextual factors that affect participants’ well-being, writing an advocacy letter to local representatives), and homework (i.e. mindfulness exercise supported by a video, logbook). Sessions’ topics are based on ACT six core processes: contact with the present moment, acceptance, defusion, self as context, committed action and values. A session is dedicated to exchanges with health professionals and advocacy of patients’ needs towards the health institutions.

The program is delivered online to support participation by geographically or socially isolated patients, or by less mobile ones. For the project, this was done via a secured Zoom platform. The program was delivered in 2020 and 2021, in the Province of Québec (Canada).

### Research objectives

The current descriptive study had two exploratory objectives: (1) run a pilot test of the program with a first group of participants; and (2) evaluate the program with two subsequent groups in a proof-of-concept perspective. We evaluated the acceptability and feasibility of the online delivery to participants; intervention proceedings and participants’ satisfaction; and short-term effects of intervention on involvement in different spheres of life, personal goals and challenges, interaction with the ROD community, quality of life and well-being.

## Method

A longitudinal qualitative design was used, within a post-positivist epistemological posture. For the pilot test (objective 1), a single interview was carried out after the intervention. For the evaluation (objective 2), a pretest (interview and questionnaire), a post-test (interview) and a follow-up (questionnaire) were carried out. The research project was approved by the University’s ethics board (number 3885-e-2020) and all participants in the groups were informed of the evaluation process. Participants in the evaluation signed informed consent forms. The involvement of participants in the program and its evaluation were illustrated in Table 1.

### Participants

Group 1, used for the pilot test, comprised four female patients who participated in the intervention. They were aged between 28 and 54 and lived with Lupus, Elher Danlos.syndrome and Lyme disease.

The sample for the evaluation consisted of 14 participants out of the 16 patients who participated in Connect-ROD Groups 2 and 3. Table 2 summarizes the characteristics of these participants.

**Table 2:** Characteristics of participants (G2, G3)

	N (%)
Gender	
• Female	14 (100%)
Age	
• Range	28 – 77
• Mean age	52.71
Family relations	
• In a relationship	7 (50%)
• With children	12 (86%)
• Average number of children	1.85
• With children living with a disability	2 (14%)
Employment status	
• Part-time employment	1 (7%)
• Unemployed	2 (14%)
• On leave of absence	2 (14%)
• On Invalidity	5 (36%)
• Retired	4 (29%)
Living environment—Locality (defined by the participants)	
• Remote area	2 (14%)
• Small town	4 (29%)
• Mid-sized town	3 (21%)
• Suburb	1 (7%)
• Large city	4 (29%)

Table 2 describes participants characteristics. All participants were female, with a mean age of 52. The majority had children and half were in a relationship. Only one participant was currently working and participants originated from a wide range of locations

Participants presented complex health conditions involving 13 different RODs (salivary gland cancer, dystonia, Ehlers-Danlos, Myalgic encephalomyelitis, Chronic fatigue syndrome or Q fever, Fibromyalgia, Gougerot-Sjögren, Lupus, Pompe's disease, BEALS syndrome, May-Thurner syndrome, Nutcrakemr syndrome, Stickler-Marshall syndrome), and many comorbidities. Each participant described between one and eight different diseases affecting several systems (motricity, cognitions, endocranial, nutritional, skeletal, neurological, sensory, etc.) and four participants received concomitant diagnoses for two different RODs. As reported by participants during the pretest interviews, these conditions are accompanied by psychological, social and material constraints that also affect their general well-being, ranging from frustration, irritability and fatigue to depression and anxiety disorders. Many reported suicidal thoughts, major bereavement, identity-related issues and social disruptions. Many have difficulty moving around, which contributes to their isolation and difficulties in working. Financial difficulties associated with issues of autonomy and mobility are also common.

The general perception of their own health status at pretest was average or low on standardised scales, such as on the PHQ-9 (m: 11.86, sd: 4.54, see Table 3), with significant variations over time. Some reported experiencing better mental than physical health and two indicated that their health globally improved recently.

**Table 3:** Mean group scores to standardized questionnaires at pretest and follow-up

Instrument	Dimensions	Improvement when scores:	Overall sample Pretest (*N* = 14) Post-test (*N* = 12) M (sd)	Distribution respects postulates	T test (paired samples)	Effect size (Cohen’s d)	Non parametric test—Wilcoxon	Effect size (r) ^a^	Non-completers (*N* = 3) M (sd)	Completers (*N* = 11) M (sd)
MHC	Global perceived mental health	↗	Pre-: 37.64 (10.89) Post-: 39.92 (12.41)	Yes	t: -0.812 (df: 11), p: .434	.23	W: .471, p: .637	.096	Pre-: 31.67 (15.01); Post-: 32.33 (17.21)	Pre-: 39.27 (9.76); Post-: 42.44 (10.42)
PHQ-9	Distress	↘	Pre-: 11.86 (4.54) Post-: 10.09 (4.57)	Yes	t: 2,901 (df: 10), p: .016	.87	W: -2.200, p: .028	-.449	Pre-: 15.00 (7.00); Post-: 12.67 (3.78)	Pre-: 11.00 (3.63); Post-: 9.12 (4.67)
PSWQ	Anxiety	↘	Pre-: 48.64 (11.13) Post-: 44.82 (11.23)	Yes	t: 1,868 (df: 10), p: .091	.56	W: -1.469, p: .142	-.299	Pre-: 47.33 (7.02); Post-: 44.67 (4.04)	Pre-: 49.03 (12.38); Post-: 44.87 (13.24)
SIDAS-FR	Severity of suicidal ideations	↘	Pre-: 3.86 (7.04) Post-: 3.33 (9.42)	No		.17	W: -.730, p: .465	-.149	Pre-: 8.00 (13.85); Post-: 11.00 (19.05)	Pre-: 2.73 (4.48); Post-: 0.78 (1.39)
S-QoL	Psychological well-being	↗	Pre-: 3.2 (0.53) Post-: 3.71 (0.72)	Yes	t: -1,486 (df: 11), p: .165	.42	W: 1.582, p: .114	.333	Pre-: 3.04 (0.38); Post-: 2.80 (1.01)	Pre-: 3.24 (0.57); Post-: 3.63 (0.68)
	Self-esteem	↗	Pre-: 4.06 (0.99) Post-: 4.58 (0.77)	Yes	t: -1,531 (df: 11), p: .154	.44	W: 1.383, p: .167	.282	Pre-: 3.53 (1.50); Post-: 3.73 (0.81)	Pre-: 4.19 (0.84); Post-: 4.42 (0.77)
	Family relations	↗	Pre-: 4.27 (1.01) Post-: 4.25 (1.01)	Yes	t: -0.571 (df: 11), p: .579	.16	W: -.476, p: .634	-.097	Pre-: 4.60 (1.20); Post-: 4.33 (1.02)	Pre-: 4.18 (0.99); Post-: 4.17 (1.03)
	Friendly relations	↗	Pre-: 4.27 (0.79) Post-: 4.25 (0.58)	No		.12	W: .462, p: .664	.094	Pre-: 4.33 (0.23); Post-: 3.80 (0.87)	Pre-: 4.25 (0.89); Post-: 4.27 (0.62)
	Resilience	↗	Pre-: 3.44 (0.97) Post-: 3.84 (0.83)	Yes	t: -1,101 (df: 11), p: .294	.31	W: 1.035, p: .301	.211	Pre-: 2.67 (1.01); Post-: 2.60 (1.31)	Pre-: 3.65 (0.88); Post-: 3.67 (0.82)
	Physical well-being	↗	Pre-: 2.66 (0.89) Post-: 2.80 (1.26)	Yes	t: -0.238 (df: 11), p: .816	.06	W: .059, p: .953	.012	Pre-: 2.33 (1.37); Post-: 2.75 (1.00)	Pre-: 2.74 (0.78); Post-: 2.65 (1.18)
	Autonomy	↗	Pre-: 4.29 (1.04) Post-: 3.89 (1.14)	No		.30	W: -.853, p: .394	-.174	Pre-: 4.42 (0.57); Post-: 4.08 (0.76)	Pre-: 4.25 (1.15); Post-: 3.94 (1.08)
	Love life	↗	Pre-: 3.39 (1.9) Post-: 3.25 (2.02)	No		.10	W: .422, p: .673	.086	Pre-: 2.50 (2.59); Post-: 2.67 (2.46)	Pre-: 3.63 (1.74); Post-: 3.42 (1.88)
Social provision scale	Attachment	↗	Pre-: 6.36 (1.45) Post-: 6.67 (0.88)	No		.45	W: 1.511, p: .131	.308	Pre-: 7.33 (1.15); Post-: 7.00 (1.00)	Pre-: 6.09 (1.44); Post-: 6.55 (0.88)
	Tangible and material support	↗	Pre-: 7.07 (1) Post-: 7.12 (0.91)	No		.16	W: .552, p: .581	.112	Pre-: 7.33 (1.15); Post-: 7.00 (1.00)	Pre-: 7.00 (1.00); Post-: 7.17 (0.93)
	Advice	↗	Pre-: 6.46 (1.05) Post-: 6.67 (1.07)	Yes	t: -1,267 (df: 11), p: .231	.36	W: 1.366, p: .172	.279	Pre-: 7.33 (1.15); Post-: 6.67 (1.15)	Pre-: 6.23 (0.93); Post-: 6.67 (1.19)
	Social integration	↗	Pre-: 5.71 (0.83) Post-: 5.62 (1.49)	No		.03	W: .970, p: .332	.198	Pre-: 6.33 (0.57); Post-: 5.33 (3.05)	Pre-: 5.54 (0.82); Post-: 5.72 (0.83)
	Assurance of one’s own worth	↗	Pre-: 5.86 (1.46) Post-: 6.21 (1.30)	Yes	t: -1,894 (df: 11), p: .084	.54	W: 1.831, p: .067	.373	Pre-: 5.33 (1.15); Post-: 5.67 (1.53)	Pre-: 6.00 (1.54); Post-: 6.39 (1.26)
Brief COPE	Active coping	↗	Pre-: 3.07 (0.73) Post-: 3.67 (0.52)	Yes	t: -2,602 (df: 11), p: .024	.75	W: 2.126, p: .033	.434	Pre-: 3.17 (1.04); Post-: 3.33 (1.15)	Pre-: 3.04 (0.68); Post-: 3.67 (0.50)
	Planification	↗	Pre-: 2.43 (0.58) Post-: 3.00 (0.45)	Yes	t: -0.761 (df: 11), p: .462	.21	W: .749, p: .454	.153	Pre-: 2.00 (0.50); Post-: 2.00 (0.50)	Pre-: 2.54 (0.56); Post-: 2.83 (0.50)
	Instrumental support	↗	Pre-: 2.71 (0.7) Post-: 2.87 (0.67)	No		.05	W: 1.147, p: .251	.234	Pre-: 2.83 (1.04); Post-: 2.00 (1.00)	Pre-: 2.68 (0.64); Post-: 3.08 (0.66)
	Emotional support	↗	Pre-: 3.05 (0.5) Post-: 3.25 (0.69)	No		.32	W: 1.033, p: .302	.216	Pre-: 3.17 (1.04); Post-: 3.00 (0.86)	Pre-: 3.02 (0.32); Post-: 3.33 (0.61)
	Expression of emotions	↗	Pre-: 3.05 (0.5) Post-: 3.25 (0.69)	No		.32	W: 1.033, p: .302	.211	Pre-: 3.17 (1.04); Post-: 3.00 (0.86)	Pre-: 3.02 (0.32); Post-: 3.33 (0.61)
	Positive reframing	↗	Pre-: 1.39 (0.66) Post-: 1.29 (0.24)	Yes	t: 0.583 (df: 11), p: .571	.16	W: -.723, p: .470	-.147	Pre-: 1.17 (0.28); Post-: 1.00 (0.00)	Pre-: 1.45 (0.72); Post-: 1.47 (0.50)
	Acceptation	↗	Pre-: 2.11 (0.59) Post-: 2.17 (0.53)	Yes	T: -0.897 (df: 11), p: .389	.32	W: .905, p: .366	.184	Pre-: 2.17 (0.29); Post-: 2.00 (0.00)	Pre-: 2.09 (0.66); Post-: 2.22 (0.62)
	Denial	↘	Pre-: 2.07 (0.43) Post-: 2.21 (0.46)	Yes	t: -1,899 (df: 11), p: .083	.54	W: 1.701, p: .089	.347	Pre-: 1.83 (0.76); Post-: 2.33 (0.28)	Pre-: 2.13 (0.32); Post-: 2.30 (0.63)
	Blame	↘	Pre-: 3 (0.83) Post-: 3.25 (0.61)	Yes	t: 0.153 (df: 11), p: .880	.04	W: .522, p: .602	.106	Pre-: 2.67 (0.76); Post-: 2.67 (1.52)	Pre-: 3.09 (0.86); Post-: 3.22 (0.56)
	Humor	↗	Pre-: 1.39 (0.56) Post-: 1.50 (0.45)	Yes	t: 0.232 (df: 11), p: .820	.06	W: -.289, p: .773	-.059	Pre-: 1.33 (0.28); Post-: 1.33 (0.57)	Pre-: 1.41 (0.62); Post-: 1.39 (0.41)
	Religion and spirituality	↗	Pre-: 2.71 (0.58) Post-: 3.17 (0.75)	No		.27	W: 1.228, p: .219	.250	Pre-: 2.33 (0.28); Post-: 2.33 (0.28)	Pre-: 2.81 (0.60); Post-: 3.11 (0.89)
	Distraction	↗	Pre-: 2.71 (0.87) Post-: 3.50 (0.89)	Yes	t: -0.654 (df: 11), p: .526	.18	W: .513, p: .608	.104	Pre-: 3.33 (0.76); Post-: 2.33 (1.52)	Pre-: 2.54 (0.85); Post-: 3.28 (1.00)
	Substance use	↘	Pre-: 1.36 (0.5) Post-: 1.33 (0.60)	No		.46	W: 1.633, p: .102	.333	Pre-: 1.00 (0.00); Post-: 1.00 (0.00)	Pre-: 1.45 (0.52); Post-: 1.67 (1.03)
	Behavioural disengagement	↘	Pre-: 2.21 (0.47) Post-: 1.87 (0.44)	Yes	t: 0.383 (df: 11), p: .709	.11	W: -.490, p: .624	-.100	Pre-: 2.17 (0.28); Post-: 2.50 (0.50)	Pre-: 2.23 (0.51); Post-: 1.97 (0.40)

^a^Effect size with r: *r* < .10 (no effect), *r* = .10 (small), *r* = .30 (medium), *r* = .50 (large). – and + indicate the direction of the effect

Table 3 presents aggregated results to all standardised instruments and subscales used in pretest and posttest for groups 2 and 3. Results are weaved with qualitative results when applicable in the result section, in order to provide an integrated analysis of interviews and questionnaire data. Table 3 also presents indicative comparisons between participants who completed the programme (completers) and those who did not ((non-completers). It indicates that non-completers experienced more initial difficulties and less positive outcomes with the programme

### Data collection instruments

For the pilot test, semi-structured interviews were done with the participants after the end of the program to gather information on content and delivery, technical issues, clarity, quality of the proposed activities, and perceived usefulness.

The evaluation was carried out with semi-structured interviews and standardised questionnaires. Interview grids for the pretest specifically included sociodemographic details, experience with RODs and healthcare systems. Pre-and post-test interviews addressed current perceptions (within the last month) of living with a ROD, engagement in various life areas, personal objectives, current challenges and obstacles, sense of control, interactions with ROD communities. Post-test interview also addressed access and use of the Connect-ROD program, experiences with it and recommendations for its improvement. Interviews lasted between 90 and 120 min.

Seven standardized questionnaires were filled out by participants (pretest and follow-up). All instructions were worded as to target the current situation of the person (over the last month). This set of questionnaires aimed at providing an assessment of the participants’ level of distress and QOL at inception in the program, as well as changes in time. The program targets distress, perception of well-being and QOL. It is therefore important to assess these dimensions within the program’s preliminary clinical assessment and to evaluate the program’s potential effects.

The Mental Health Continuum (MHC–SF) comprises 14 items rated on 6-point Likert scales and measures perceived general well-being as a distinct dimension from mental illness. Global mental health can be perceived as poor, moderate or flourishing, with scores ranging from 0 to 70. Mental health is perceived more positively when score is higher. It was validated in French with a three-factor structure presenting a high level of internal consistency (α ranging from 0.79 to 0.90 (emotional, social and psychological well-being) [36].

The Patient Health Questionnaire-(PHQ-9) assesses the frequency of depressive symptoms on nine Likert scales (four points). A cut-off point of 10 is usually used to identify individuals at risk of depression. It has been validated in French and shows high internal consistency (α = 0.86) and a unifactorial structure [37, 38].

The Penn State Worry Questionnaire (PSWQ) measures symptoms of stress and anxiety on 16 items, rated on 5-point Likert scales. A score between 16 and 52 indicates a low risk, while a score of 53 and above indicates a high risk for a general anxiety disorder. It was validated in French, presents an unifactorial structure and shows strong internal consistency (α = 0.92) [39].

The SIDAS-FR, a short scale with 5 items rated on an 11-point Likert scale, assesses the severity of suicidal ideations. It has been validated in French and shows high internal consistency (α = 0.83) and a unifactorial structure. Cut-off scores of 13 and 20 indicate moderate and high severity of suicide ideations and guide orientation to appropriate professional suicide risk assessment [40]. Suicidality is rarely assessed in the context of ROD [18] and a recent study showed that at least 36% of patients experiences suicide ideation [41]. It is therefore important to include a measure of this dimension in the current study.

The Schizophrenia Quality of Life questionnaire (S-QoL) is based on 41 items rated on a 6-point Likert scale and assesses eight dimensions of quality of life. The score ranges between 0 and 100, with higher score indicating a higher QOL In French, it shows satisfactory internal consistency for each dimension (α between 0.72 and 0.92) and is sensitive to change over time [42].

The Social provision scale – 10 (SPS-10) is made up of 10 items rated on a four-point Likert scale, that assesses five dimensions of social support, and has been validated in French [43]. A higher score indicates stronger social support on each dimension. It presents a strong concurrent validity with the original 24-item scale, and a strong internal consistency (α = 0.880).

The Brief-COPE is a 28-item scale measuring 14 dimensions of coping styles on four-point Likert scales. The score for each dimension varies between 2 and 8 and a higher score indicates a stringer use of this dimension of coping. It has been validated in French with a 14-factor structure [44].

### Procedure

Recruitment was performed via an ad placed on social media and in network groups for AP-ROD. These varied recruitment methods facilitated the inclusion of a diversified group of patients with different RODs and from various regions. Included participants were recruited from the ad (*N* = 13), word-of-mouth (*N* = 2), pamphlets in a medical clinic (*N* = 1) and direct email by a peer-led support group administrator (*N* = 2). Participants had to have a diagnosis for a ROD and all types of ROD were included.

The pilot test (Group 1) took place from September to December 2020 and was used to assess overall feasibility and test the online delivery process. Group 1 comprised *N* = 4 participants and there was one attrition to the program due to medical reasons.

The evaluation phase was performed with two groups of participants, Group 2 (January to April 2021, *N* = 6) and Group 3 (May to July 2021, *N* = 10).

The program was delivered by trained psychology graduate students who had received their primary clinical training, including training in ACT, and were supervised by an ACT specialist (coauthor MR). Co-facilitators were psychology undergraduate and graduate students.

As to interfere as little as possible with the program’s process, recruitment for the research project was performed after inception of participants into the program. All participants to the pilot project were invited to also participate in the evaluation, but participation in the evaluation was not a condition for joining the group.

Interviews were scheduled to suit participants’ availability, carried out via Zoom, audiotaped and transcribed. Questionnaires were sent by email to participants.

### Analyses

N-Vivo 12 was used for coding interview contents. Thematic analyses were performed by coauthors MER and JZ (graduated students in psychology, trained in qualitative analysis), supervised by coauthor CB, and using Braun and Clarke [45]. MER and JZ familiarized themselves with the transcribed material. Extensive exchanges helped produce a preliminary coding grid based on deductive (what participants say about our topics of interest) and inductive (what emerges from participants’ discourse) approaches. Preliminary coding of a subsample of interviews was performed by MER and JZ. Discrepancies in coding were discussed to improve the coding grid. The revised coding grid was the applied to the entire corpus of data. A synthesis of the codes was written, and relevant quotes identified. The syntheses were discussed amongst team members before the results were written by CB.

Standardized questionnaires were coded and associated with each participant’s interview and analyzed along with the interview data. An overall descriptive portrait of the variations in scores is also summarized in Table 3, including a T-test of Student (parametric test, when data respects postulates, effect size with Cohen’s d) and Wilcoxon test (non-parametric test, effect size with r, for all variables) to provide an indication of the potential quantified effects of the program. Because of the structure of the project, the sample size was too small for statistical analyses to be significant, but with effect sizes (“r”), they can provide a global direction to the results.

## Results

A narrative structure was chosen to report results in a chronological order, from program inception to short term effects. It combines results from the thematic analyses of interviews and, when specified, from descriptive analyses of standardised questionnaires presented in Table 3 to address each evaluation objective successively.

### Pilot test

Participants in group 1 provided the name of the program and recommendation for its improvement. Minor adaptations to the content and format of the program were performed after the pilot study, based on this feedback. Specifically, the program was changed from 8 to 10 sessions and more emphasis was placed on sharing opportunities between participants.

### Acceptability and feasibility of the program and online delivery

#### Reasons to engage in the Connect-ROD program

Participants were motivated by the need to receive psychological support (*N* = 5), to have tools to get better (*N* = 3), to live better with their ROD (*N* = 5), to be able to share their experience with others (*N* = 3), to vent (*N* = 5), to reduce isolation (*N* = 2) or to better organize their daily life (*N* = 1).

*“I was going through a very difficult time with respect to accepting the disease. I thought that it would be a plus for me to be able to live this experience with other people. I knew I had to move on to something else because I was in a vicious circle in my head. I had difficulties getting over the ideas I had about the disease. Then I knew that I had to work with it. Then the group allowed me to do that. When I saw that, I said to myself:’Well, I’m going to be able to move forward a little more. I’ll be able to get some tools to help me.’ That’s why I signed up in the first place.” (P31).*

Three participants mentioned specifically that participating in the group was important to them because it contributed to the recognition of ROD in the community.

#### Accessibility of the program

Participants found that communicating about the program in online patient groups was promising. However, the multiplicity of these groups and the fragmentation of these networks can become a barrier to communication. To address this, participants suggested that information about the program be disseminated to health services, community organizations and directly to health care professionals.

#### Accessibility of program tools

Once enrolled in the program, participants felt they had easy access to the clinical and communication tools available to them. Homework and materials used during the activities were sent by email the day after the activity and participants appreciated this modality. Sharing videos by email or during the sessions was also successful.

#### Online groups

Participants (*N* = 13) noted the unique value of holding the group online, to bring together people living in different and remote areas and to allow participation by people with very low energy levels or mobility issues. For example, sometimes participants attended the group from their beds. However, holding the group online was problematic for one participant who did not have a private space in her home and therefore was reluctant to discuss sensitive topics for fear of family members overhearing her.

A few participants (*N* = 3) felt that the fluidity of exchanges would have been different in a face-to-face setting, particularly because of the way speaking turns were organized. While one participant underlined that she had the impression that the human warmth was not the same as in face-to-face, another was, on the contrary, rather surprised to see that the exchanges could be so emotional at a distance and that she did not feel any barriers associated with the online modality.

*“I was really surprised, probably because there are not many of us. We talked about it in the last session that it should stay that way. Because even if it’s the screen, there are only a few of us, that’s what we said to each other, listen, we had some pretty eh… emotional exchanges and the screen didn’t create a barrier at all, I didn’t feel that it created a distance or anything, on the contrary, we didn’t see it and it allowed us once again to see different types of people in different places.” P23.*

#### Technical issues

No technical issues were reported by participants that would have hindered their participation in the program. The Zoom platform was perceived as easy to use and participants adapted quickly to the online modalities. Technical assistance was available from the co-facilitator but was seldomly used.

### Intervention proceedings and participant satisfaction

#### Attrition

Three participants abandoned the program, but all completed the research protocol. Reasons for dropping out were reactions to some very emotional comments made by participants (*N* = 2) and medical issues (*N* = 1). In addition, one participant indicated that the program did not meet her needs as she felt she was further along in her journey than other participants. Participants who completed the program (*N* = 11) participated in 8 to 10 sessions.

#### Facilitators’ work

All participants indicated that they appreciated the work of the two clinicians and of the co-facilitators delivering the program. Qualities highlighted by the participants were the respect for group members and their pace, the ability to create a climate of trust and to encourage participants to express themselves, listening skills, empathy, openness, flexibility, and the ability to explain concepts clearly. In Group 3, the clinician used examples and metaphors from her personal life, which was also very much appreciated.

*“I thought she seemed approachable and uh, was easy to talk to, and even she shared her personal experiences, and you know, it was fun, I liked that, yeah. I felt like she was really part of the group, it wasn’t like someone outside of the group who was in charge, it was like she was with us, even though she didn’t have a rare disease.” (P35).*

The only downside was that one participant found some of the explanations and interventions too theoretical when it came to the concepts associated with ACT.

All planned sessions were completed in both groups, although some activities were turned into post-session homework to allow for prolonged discussion among group members during the sessions.

#### Feeling of engagement in the program

Most participants cited intrinsic sources of motivation to remain engaged in the program, and applied strategies to maintain their participation, such as limiting their activities for the day to conserve energy. A few program features were identified as supportive of participants’ engagement, such as group dynamics, sharing, positive atmosphere among participants, structure, topics and facilitators.

However, one participant indicated that the sharing of difficult experiences and very negative emotions by others interfered with her engagement in the program and caused her to leave the group.

#### Satisfaction with the program

Overall satisfaction was high, as expressed in post test interviews. However, participants brought nuances to their appreciation that need to be considered.

The heterogeneity of the participants (in terms of type of illness and position in the trajectory of living with the illness) can be seen as a limitation. Some people reported feeling frustrated at hearing “new patients” go through the same steps they had taken years before, felt they were giving more support than they were receiving, or perceived too many differences in the experiences of participants living with different levels of disease and limitations. However, others saw this heterogeneity as a strength of the program, allowing them to meet people who were different but who shared similar experiences.

There is a great need to share one’s experience and to discuss it, and the program does not necessarily give enough sharing space between structured activities. There was some tension among some participants, as some wished for the program to run its course as planned (that the activities be carried out and sufficiently explained) while others expressed the desire that everyone be heard according to their needs.

The length and pace of the program could be adapted to the specific needs of different participants. For example, some people feel better in the morning, others in the afternoon, the 2-h sessions are too long for some people and others would like to be able to progress more slowly.

The metaphors, visual tools, and audio recordings were all appreciated and helped to reinforce understanding and retention of ideas, messages, and strategies.

Most participants enjoyed the exercises and found them relevant. However, some found it difficult to do homework regularly due to lack of motivation (*N* = 2), visual limitations (*N* = 1), or because they had difficulty understanding them (*N* = 2).

Inviting a health care professional involved in the management of RODs to discuss with the groups allowed participants to reflect on their relationship with the medical system and prepare specific questions and information that would be helpful in reinforcing their sense of control. It was suggested that more varied professionals be involved to support the exchange between patients and the health care community.

#### Appreciation of specific activities

All activities seemed to be similarly well appreciated. However, some appeared more difficult, like naming emotions or the recurrent relaxation session. The activities that were the most appreciated were “the wheel of emotions,” the metaphors presented in video format, especially “quicksand” and “the bus,” meditation, and relaxation.

*“The videos they stay with you. Even if we had the theoretical level, but the videos I find that it remains, because it is printed in my head, it is printed at the visual level, at the level of memory” (P22).*

Exercises and homework were also very much appreciated because they encouraged thoughts and reflexivity.

*“The exercises make you think and target the important emotions. In any case, I find that it helped me to center myself a little more than [sigh], you know, it happens to me and [sigh] there is a kind of explosion of emotions, you don’t really know how to manage that, but I find that it succeeded a little bit in centering the matter and in settling down a little bit” (P35).*

Several participants indicated that the activities and tools help them directly in their daily lives and that they use them regularly.

*“I’ll give you an example, you know, I just tried to go out for an errand and I was in pain, and normally I would have been angry, but instead I remembered, ‘Okay, take a deep breath, go back to now, the present, it’s okay. That’s all stuff I took from the program.” (P22).*

It was suggested that a module be added to the program on communicating with loved ones about the disease and how to achieve a balance between managing others’ reactions and setting boundaries.

#### Group structure

The majority of participants appreciated the exchanges during the group sessions. Many mentioned that the group provided a warm atmosphere of sharing, mutual aid and commitment. It also allowed them to feel less isolated, to normalize their reactions to the disease and to find a common experience with others, despite the differences between participants.

A lot of sharing was done during the sessions and exchanges between participants were important, which seemed to suit a majority of participants. According to the participants, the group dynamic was “natural” and encouraged rich exchanges.

#### Satisfaction of participants’ objectives

Most of the participants indicated that the program met their needs, by learning to better manage their emotions, assert themselves, strengthen their sense of competence, acquire tools to manage daily life, gain motivation to engage in projects, and develop acceptance in relation to the disease.

*“My objectives were met by the techniques and then by the support provided by the group. Then my objective was to feel better about my mental condition versus the disease. And I think that I have come a long way in a short period of time with the group, so I think that my expectations have been totally met. It’s not 100%, but I feel like I’m on the right track.” (P31).*

On the other hand, some (*N* = 3) felt shortcomings associated with the lack of depth around certain themes (e.g., putting more emphasis on hopelessness instead of addressing all the emotions), their lack of psychological availability to participate (e.g., feeling overwhelmed by the negative emotions of others) or their lack of physical availability (e.g., hospitalization).

### Effects of the intervention

The effects of the program are very difficult to isolate from the fluctuating nature of the participants’ life situation as it relates to the ROD they live with. For example, one participant indicated that she was experiencing more coping and social difficulties at post-test, related to a hospitalization she underwent during the project. In a case like hers, the decrease in well-being indicators cannot be linked to the program and may have been greater if she had not participated in the program. An in-depth qualitative analysis of the perceived impact of the program on participants’ functioning processes was therefore conducted. Only those results are presented that associate program processes with changes described by the participants.

Table 3 shows participants’ mean scores on the standardized tools used at pretest and post-test, including T-tests and effect sizes as indicators of program effects. Details are presented alongside qualitative data when applicable in the sections below. Table 3 also shows mean scores for program completers (*N* = 11) and non-completers (*N* = 3). Overall, completers of the program seemed to end up with better scores of well-being and improved more than non-completers.

#### Personal objectives

Setting attainable goals on one’s own is an important part of the recovery processes supported by the Connect-ROD program.

*“I’ll tell you that, that’s been a big part of my journey with the group. Because before that, I was always in denial. Then if I had a challenge, I would do it no matter what. And I didn’t respect myself in that. Since then, I’ve been trying to challenge myself, to set healthier goals. Then I manage to do more. Maybe it wasn’t every day, but I’ll tell you that for the most part, since the group, I’ve been able to do more. I think it’s great.” (P31).*

Several perspectives were stated, reflecting different processes related to the program among participants. One person indicated that she became aware of the importance of setting attainable goals, but still felt limited by the disease and associated lack of energy and therefore felt unable to do what she wanted to do. This mindset remained over time. Many said that they had long ago learned to set short-term goals “because it’s important” based on the energy fluctuations associated with the illness, and to create space for action within those limits to enhance their sense of purpose and maintain good mental health (*N* = 7). They did not change their perspective with the program. Finally, one group developed, as a result of the program, a habit of making a special effort to set realistic goals or make adaptations to achieve them (*N* = 6).

Most participants had difficulty projecting positively into the future and preferred to think about their lives in the very short term, with some even refusing to think about it at all. However, a small number of participants (*N* = 3) were able to develop a more optimistic perspective on their future, imagining themselves living better with the disease, accepting the chronicity of the disease, having access to better medical services. Others made new plans in relation to their social network (*N* = 4) in order to increase the quantity and quality of their relationships.

Beyond these personal projections, some of them have projects related to activism (*N* = 3) aiming at the creation of patient associations, systemic changes at the government or health system levels. They decided to get more involved in order to change the world in which they live and the program seemed to contribute to their renewed motivation.

*“That’s systemic too. That’s why my goal is to work on this problem. That’s why I do advocacy, as they say, that I get involved a lot, hoping that it will change these ways of doing things, that it will change perceptions, *etc*. That’s my big goal.” (P30).*

#### Self-efficacy and sense of competency

The program seemed to help build capacity and confidence to achieve goals for many participants.

*"I think I’ll just have to be gentle with myself. That the 7 or 8 (out of 10 on self-efficacy) might be there, but maybe not right away. It’s going to come eventually with the integration of the tools." (P26).*

Others indicated that they had gained confidence by having a space to learn to express their experiences and needs, and to feel legitimate to talk about their illness in other contexts and with their loved ones.

The sense of competence was also reinforced for two participants through the acquisition of new capacities to support other AP-ROD within the program and for two others by learning to recognize and accept their limitations, which helps them to adapt more effectively.

#### Sense of control

Most participants felt in control of different areas of their lives, but not their bodies and symptoms and this has not changed.

*‘Makes me feel in control really more of my family, and emotional mode. But otherwise it’s rough every day… […] Because I feel like I have no control over my body. Because my body, if it decides that my leg hurts, I can’t do anything about it. It’s there, then I have to deal with it.’ (P31).*

However, two participants explicitly stated that the program helped them gain control over their attitude and knowledge about the disease.

Part of the sense of control can be expressed through their ability to influence others and what happens around them. Six participants reported that their feelings of being able to influence those around them changed in nature (different areas of influence were added, such as writing a group letter to a local politician) and in intensity (being more heard in their circle of friends) during the post-test.

#### Coping mechanisms

On the Brief COPE (see Table 3), overall scores are higher for active coping, emotional support, expression of emotions and blame at pretest than for other coping dimensions, and participants did not indicate that they made intensive use of a large range of coping mechanisms. At post-test, the use of planification and distraction increased for most participants. There was also a reduction of the utilization of behavioural disengagement. Other coping mechanisms did not vary between pretest and post-test in most participants. Non-completers seemed to benefit from less instrumental and emotional support, experienced more denial, were using distraction less and more behavioural disengagement at post-test, when compared with pretest.

#### Symptoms management

Half of the participants reported changes in the way they perceive their symptoms, even if these changes are neither linear nor constant. They learned to live with them, to listen to their bodies, to accept the necessary limitations and to slow down.

*“And I’m starting to accept…. I’m not starting, I’m accepting the fact that now I have shortcomings with respect to certain things, certain actions that I’m not able to do anymore. For example, I have a cane. I didn’t use it before. I was like I don’t need it, then I was hurting myself. Now I take my cane, and I find the benefits important, that it encourages me to take my cane again. All the journey that it has done, for me, it is beneficial. It’s less pain, less mental conflict, less rage” (P31).*

Those who did not experience a change were divided equally between those who already had ways of constantly adapting to their symptoms and their evolution and those who still felt as much difficulty and frustration with their situation, and whose perception patterns had not changed.

#### Social support

Participants experienced generally quite high support on the various subscales of the social provision scale (Table 3) at pretest and most participants experienced an increase in their social support at post-test, except on social integration and tangible and material support. However, non-completers experienced decreases in their social support at post-test.

The majority of participants were already receiving social support prior to the program and this support did not change with the program. However, some participants indicated that they had improved their ability to communicate their needs to their loved ones, which increased the support they received (*N* = 4). On the other hand, participants who were very isolated before the program (*N* = 2) remained so.

The quality of social relationships also improved for half of the participants. They were able to distance themselves from certain people and expand their network. They became more comfortable talking about their illness with their loved ones to explain their social difficulties and felt more accepted and able to interact.

The size and density of the participants’ social network do not seem to have changed as a result of participation in the program, although some have become aware of the importance of strengthening and diversifying their network.

#### Social engagement and connecting with ROD communities

Participants created a Facebook support group “Connect-MRO, next steps” independent of the program developers and all joined to continue social support activities after the program ended. Several became involved in managing this group and integrating new members. From information we were able to gather, the group met once a week for at least six months at the same hour the program used to be. In addition, two participants mentioned that through this group, a letter was sent to governmental bodies in relation to RODs and the development of the rare disease policy that was being created.

Some participants were able to share information and resources on various patient organizations and processes for accessing health services. Others felt encouraged to make greater use of the resources they already knew about.

Several reported benefiting from being able to interact with other AP-ROD with whom they could share a common experience (*N* = 4).

“It’s good to see people who are going through the same thing as you. The same difficulties. Then we can talk…then we can talk about it openly.” (P34)

Others were able to feel less isolated during the COVID-19 lockdowns (*N* = 2).

#### Acceptance of the emotional experience associated with the disease

A few participants (*N* = 4) reported being more able to accept their illness and limitations. One participant described going from rage, anger, feelings of injustice and denial about her illness to acceptance, and gave the example of the accommodations she made in her daily life to continue her activities within the limitations of her illness. Rather than depriving herself of participating in activities she enjoys, she now considers doing them with a wheelchair since another participant in a wheelchair shared her experience. Another participant reports that she has regained some control over her life choices rather than letting the disease determine them. Others did not experience changes in this area.

#### Psychological well-being

Participants report that the program contributed to improved mood and reduced distress.

*“Because that’s the real [participant’s name] that I know. She’s not the one doing nothing and waiting for whatever. I was talking about it with my son. I wasn’t depressed, I was lethargic. I was looking for myself. […] I regained my independence. I got my momentum back. The real [participant’s name] who fights, and who is in all kinds of business, and who moves!”—P27.*

Participants who completed the program (*N* = 11) seemed to experience more improvement in their psychological well-being than those who did not (*N* = 3, see Table 3).

Compared to non-completers, program completers generally started the program with better perception of their mental health (MHC), fewer distress symptoms (PHQ-9), and fewer suicidal ideations (SIDAS). At post-test, completers seem to have improved more than non-completers on MHC, PHQ-9, and PSWQ scores (Table 3).

Finally, seven participants (*N* = 7) expressed suicidal ideations at pretest (SIDAS-fr), including one with a moderate suicide attempt risk and one with a high suicide attempt risk. No additional participant expressed suicidal ideations at post-test and suicidal ideations receded for three participants, including the one with moderate risk of suicide attempt (all completers, Table 3). The risk level remained high with the high-risk participant, who experienced a hospitalization, did not complete the program and received additional support from suicide prevention services during the course of the program. Interestingly, distress and anxiety were lower in this participant at post-test.

#### Quality of life

Participants generally indicated moderate levels of QOL (S-QoL, see Table 3) on all subscales, and low for physical well-being at pretest. Scores generally varied a lot for participants between pretest and post-test, except for resilience that remained stable for almost all of them. Variations in scores seem to be related to personal life events such as an unforeseen hospitalization or a breakup, and are difficult to associate with the program. However, non-completers’ scores at post-test indicated less improvement than completers.

## Discussion

Adult patients living with various RODs experience common psychosocial difficulties and needs that can be considered in a trans-diagnosis manner. This project aimed to develop and evaluate an online therapeutic group intervention for AP-ROD to address theses patients’ needs. Connect-ROD is a manualized 10-week program made up of 2-h sessions and delivered online by a team of trained ACT facilitators and students or peer cofacilitators. It is supported by a set of ACT and community psychology facilitation tools. It is designed to be delivered online but could be adapted for face-to-face implementation. To our knowledge, Connect-ROD is the first published program of this type.

This project performed an initial evaluation of crucial aspects of the program. It analyzed the delivery and short-term effect of Connect-ROD, implemented in a context that could resemble a naturalistic setting as closely as possible. It is one of the rare studies to include only AP-ROD, although the diseases themselves were varied. Acceptability and feasibility were satisfactory with low attrition rates during the program (18.7%) and the study protocols (0%), and a high level of engagement from the participants in the program and with the group. Online delivery was well received and appreciated, encouraging participation from isolated and less mobile individuals. Intervention processes took place as planned, and program material was usually accessible and used. The approach adopted by the facilitators and their interventions were well appreciated and their ability to adapt the rhythm of activities to allow room for exchanges between participants was underscored. The clinical approaches and associated activities were deemed relevant and useful by participants. Satisfaction was high, although different participants saw group heterogeneity as either a strength or a limitation. There also was some tension among participants between a need to share experiences and emotions and a need to complete the planned activities. However, participants stated that their objectives in participating in the program were met.

The effects of the program were approximated via the perceptions of participants on several key proximal factors (personal objectives, self-efficacy, sense of control, coping, symptom management, social support, and social engagement) that could influence acceptance of the experiences and emotions associated with the disease, psychological well-being and quality of life. Within two to four weeks after its completion, the 10-week program had the following effects: very few participants experienced an improvement in quality of life; an average number of participants experienced a moderate effect on personal objectives, sense of control, coping mechanisms, symptom management, acceptance of the emotions associated with the disease, distress; and a majority of participants experienced an important effect on self-efficacy, social support, social engagement and connectedness with ROD-communities. Non-completers experienced less positive changes than program completers. We conclude from this data that this program is promising in terms of supporting AP-ROD as it seems to have an impact on elements associated with potential improvement of well-being in these groups. Our small sample did not allow us to identify characteristics of the participants that could affect the program’s effects.

### Key issues related to program implementation and effects

In light of this pilot project, of our preliminary needs analysis of AP-ROD [29], and of current scientific literature [25], a few key issues arose in developing and evaluating Connect-ROD.

Conducting an online group intervention raised issues of equity in access. Online groups increase participation of geographically isolated or less mobile patients [18]. Eight Connect-ROD participants came from areas where the program could not have been provided face-to-face in the current context, and less mobile participants could also not have been included. Furthermore, patients who experienced physical deterioration could maintain their involvement throughout the program because they could do so from their bed. However, online programs can present with accessibility issues that may increase iniquities. Participants must have access to electronic equipment and reliable high-speed internet connections that are costly and may not be generally available. Computer literacy and a generally higher level of education are required since participants were informed of the program online, had to be able to connect to the platform, to receive emails and to read written documents. Although this issue was not directly raised within the project, it is important to be aware of literacy issues to ensure equity in the delivery of this type of program. As was noted by one of our participants, the access to private personal space may also be an issue for accessibility to the online intervention program and may limit some participation. Finally, sensory disabilities can also hinder participation, as was the case for a visually impaired participant who received some help from a peer to read the program material. Encouraging disclosure of sensory impairment at inception interview and adapting program modalities would increase accessibility.

A choice was made to include patients dealing with various ROD in the group, because they share common psychosocial needs [3, 46] and experience a lack of resources adapted to their situation [47]. However, programs usually involve patients with the same disease, but the recent review by Bardon, Guillemette [18] did not identify any specific scientific or clinical argument to support either choice. Results from the current study indicate that mixing patients with various RODs did not negatively affect program processes and outcomes, and participants underscored the richness of sharing their experiences with patients living with different diseases but similar needs. It brought an overarching sense of community and common goals to participants, that manifested in the writing of group letters to members of parliament to contribute to the pressure to produce a national strategy for rare diseases.

However, in mixing participants in the groups, specific attention should be paid to the following issues. Including participants at different stages of the same disease could have iatrogenic effects for those who have fewer or milder symptoms, who might experience high anxiety when meeting patients with more advanced problems caused by the same disease, but also for patients who have been living longer with the disease and so feel they provide more support to “new” patients than they receive from the group. This issue should be discussed at inception with potential participants, especially those who experience high levels of emotional distress and support needs.

The inclusion of suicidal participants in intervention and in research protocols is an ongoing concern [48]. Because the clinical and research teams for Connect-ROD are well trained in suicide prevention and are used to carry out research projects with suicidal participants, it was decided to include participants with suicidal behaviour (SB) in order to improve equity of access to needed support for these vulnerable but often overlooked patients. To our knowledge, the presence of SB has never been assessed in the context of the evaluation of intervention programs with AP-ROD and the high incidence of these SB in our sample (*N* = 7/ 14 participants) shows it is a critical issue that should be considered in program processes and outcomes. AP-ROD with SB fully participated in the program and were able to share their experience with SB, while feeling supported by both the facilitators and the groups. No contagion effect was observed within the groups, with no emerging SB occurring in non-suicidal participants, which concurs with previous studies on the safety of including suicidal patients in group interventions [49]. One participant felt uncomfortable with the high level of distress shared by others and left the group, acknowledging she was not emotionally available enough to participate in the process. Exposition to others’ emotional distress and levels of anticipated comfort with it should be discussed at inception interview.

### Limits of the current study

This study must be understood within the constraints of its methodological limits. It is based on an exploratory small-scale short-term pilot project and a convenient sample of 14 participants recruited from the community and participating in two groups. This means that it was impossible to know what the actual visibility of the published ads on social media was and whether the intended target public was really reached. From a methodological point of view, statistical analyses were not appropriate to assess the impact of the program on participants’ well-being and in depth, qualitative analyses were prioritized. Issues of generalization are therefore to be considered.

The absence of a control group limited the scope of conclusion that could be drawn on the effect of the intervention. However, with these patients, who experience very important variations in well-being due to the impact of their disease on their daily life, it is important to use creative ways to assess the effects a program such as this one can have. In-depth reflexive interviews with participants were a promising avenue explored in this project and could be used successfully in a larger scale project.

The study needs to be scaled up within a mixed method design to better understand the mid- to long-term impacts of the program within the context of health services continuum, and in interaction with other services received by patients.

## Conclusion

Connect-ROD has been developed to be easily implemented and administered in community settings by trained ACT facilitators. Based on the results from the pilot study, we conclude that the program could be a relevant addition to integrated patient-centred community-based care for AP-ROD. However, large-scale, long-term evaluation is needed to consolidate these preliminary findings.

## Data Availability

The datasets generated and/or analysed during the current study are not publicly available due to the fact that individual participants and situations could be identified in interview transcripts, but are available from the corresponding author on reasonable request.
